# Bacteria in Peanut Nodules Under Herbicide and Non-Herbicide Management: Isolation, Identification, and Screening of Plant Growth-Promoting Traits

**DOI:** 10.3390/microorganisms14051004

**Published:** 2026-04-29

**Authors:** Heytor Lemos Martins, Natália Sarmanho Monteiro Lima, Luís Angel Chicoma Rojas, João Francisco Bronhara Pereira, João Francisco Damião Zanqueta, Cristina Veloso de Castro, Jhansley Ferreira da Mata, Eduardo da Silva Martins, Camila Cesário Fernandes Sartini, Eliana Gertrudes de Macedo Lemos, Pedro Luís da Costa Aguiar Alves

**Affiliations:** 1 Department of Biology, School of Agricultural and Veterinary Sciences, São Paulo State University (UNESP), Jaboticabal 14884-900, SP, Brazil; natalia.sarmanho@unesp.br (N.S.M.L.); joao.bronhara@unesp.br (J.F.B.P.); jf.zanqueta@unesp.br (J.F.D.Z.); pl.alves@unesp.br (P.L.d.C.A.A.); 2 Molecular Biology Laboratory, Institute for Research in Bioenergy (IPBEN) and Multiuser Laboratory for Large-Scale DNA Sequencing and Gene Expression Analysis (LMSeq), Faculty of Agricultural and Veterinary Sciences, São Paulo State University (UNESP), Jaboticabal 14884-900, SP, Brazil; luis.chicoma@unesp.br (L.A.C.R.); camila.c.fernandes@unesp.br (C.C.F.S.); eliana.lemos@unesp.br (E.G.d.M.L.); 3 Department of Agricultural, Livestock and Environmental Biotechnology, School of Agricultural and Veterinary Sciences, São Paulo State University (UNESP), Jaboticabal 14884-900, SP, Brazil; 4 Department of Agricultural and Biological Sciences, State University of Minas Gerais, Frutal 38202-436, MG, Brazil; cristina.castro@uemg.br (C.V.d.C.); jhansley.mata@uemg.br (J.F.d.M.); eduardo.martins@uemg.br (E.d.S.M.)

**Keywords:** *Arachis hypogaea*, nodulation, imazapic, *Bacillus*, endophytes, bioinoculant, 16S rRNA

## Abstract

Peanut (*Arachis hypogaea* L.) forms root nodules that host microbial communities influencing plant nutrition and stress tolerance, and herbicide use may act as an environmental filter altering the cultivable nodule microbiota. This study isolated and characterized bacteria from peanut nodules collected in fields with and without imazapic application in Jaboticabal, São Paulo, Brazil. Eight isolates were obtained, and one hemolytic strain was excluded after pathogenicity screening. Based on 16S rRNA gene sequencing and phylogenetic analysis, the isolates were identified as *Bacillus aerophilus*, *Bacillus inaquosorum*, *Bacillus subtilis*, *Bradyrhizobium yuanmingense*, *Burkholderia lata*, and *Rhizobium tropici*. Nodules from herbicide-treated plants yielded exclusively *Bacillus* spp., whereas those from non-treated plants showed greater taxonomic diversity. Molecular screening detected genes associated with biological nitrogen fixation (*nifH*) and nodulation (*nodA*, *nodB*, *nodC*, *nodD*), indicating potential functional capacity. In greenhouse assays, the isolates showed strain-dependent effects on early plant development, with pronounced responses in root growth and nodulation. *Burkholderia lata* and bacterial consortia enhanced root development and nodulation, with performance comparable to the commercial inoculant SEMIA 6144. Herbicide management shapes the cultivable nodule microbiota, and selected isolates show potential as bioinoculants for peanut production systems.

## 1. Introduction

Legumes play a central role in sustainable agricultural systems due to their symbiosis with nitrogen-fixing bacteria, which contributes to nutrient cycling and soil fertility. Although root nodules were traditionally regarded as exclusive niches for symbiotic rhizobia, molecular studies have demonstrated that they are complex microenvironments harboring diverse and metabolically active bacterial communities. These nodules function as structured microbial habitats, where interactions such as competition, cooperation, and metabolic complementarity may influence community assembly and function. This is particularly relevant because nodule-associated non-rhizobial bacteria may affect plant growth, nutrient acquisition, and stress responses; however, their composition and functional significance remain poorly understood in many legume systems [1,2].

Metagenomic and 16S rRNA gene-based studies have identified non-rhizobial endophytes (NREs) as common members of the nodule microbiome, in some cases representing a substantial proportion of the bacterial community [2,3]. Among them, genera such as *Bacillus*, *Burkholderia*, *Pseudomonas*, and *Enterobacter* are frequently associated with plant growth-promoting functions, including phytohormone production, nutrient solubilization, biofilm formation, and abiotic stress tolerance. [4,5]. In addition, evidence suggests that the coexistence of rhizobia and NREs may occur in a synergistic manner, enhancing nodulation efficiency and biological nitrogen fixation, particularly under adverse environmental conditions [6,7].

Although increasing evidence highlights the diversity and potential functions of bacteria associated with legume nodules, less is known about how agricultural management practices shape these communities. Among these practices, herbicide application may act as an important environmental filter by imposing selective pressure on soil microbiota and favoring microorganisms with greater physiological plasticity and tolerance to chemical stress [8]. However, the extent to which this selective pressure influences the physiological, metabolic, and genetic characteristics of bacteria isolated directly from nodules remains poorly understood, as do its implications for plant growth promotion and the stability of the rhizobium–legume symbiosis [9].

In this context, we hypothesized that herbicide management alters not only the composition but also the functional potential of the cultivable bacterial community associated with peanut nodules, by selectively favoring microorganisms with greater tolerance to chemical stress and distinct plant growth–promoting traits. Accordingly, this study aimed to evaluate these effects through bacterial isolation, molecular identification, physiological characterization, and plant inoculation assays.

## 2. Materials and Methods

### 2.1. Experimental Area

The experiment was conducted during the autumn–winter growing season at the experimental field of the Weed Science Laboratory and at the Laboratory of Plant and Microbial Biochemistry, São Paulo State University (UNESP), Jaboticabal, São Paulo, Brazil (21°14′39.83″ S, 48°17′56.84″ W; 606 m a.s.l.). The treatments consisted of peanut crops cultivated under two management conditions: with and without the application of the herbicide imazapic (Plateau^®^).

For each treatment, eight plants were randomly sampled at the R5–R6 growth stage. All nodules were collected from each plant, resulting in a total of 24 nodules in the non-treated condition and 15 nodules in the herbicide-treated condition. The lower number of nodules observed reflects the sampling stage, as nodulation is typically reduced during reproductive stages compared to vegetative growth. Each plant was considered an experimental replicate for subsequent analyses.

The chemical analysis showed the following results: pH (CaCl_2_) = 5.6, OM (organic matter) = 16 g dm^−3^, P (phosphorus) = 6 mg dm^−3^, K (potassium) = 3 mmolc.dm^−3^, Ca (calcium) = 33 mmolc.dm^−3^, Mg (magnesium) = 8 mmolc.dm^−3^, H + Al (potential acidity) = 20 mmolc.dm^−3^, SB (sum of bases) = 44.1 mmolc.dm^−3^, CEC (cation exchange capacity) = 63.9 mmolc.dm^−3^, V (base saturation) = 69%. The results of the granulometric analysis showed the following: clay = 53%; silt = 21% and sand = 26%. The peanut cultivar used was IAC 503. 

### 2.2. Isolation and Cultivation Conditions of Microorganisms

Roots were collected and surface-disinfested following the method described by Araújo et al. [10], with modifications, to remove epiphytic microbial communities. The procedure consisted of immersing the roots in 95% ethanol for 1 min, followed by immersion in 2.5% sodium hypochlorite for 4 min, and finally in 70% ethanol for 30 s. After disinfestation, the roots were rinsed thoroughly with running water followed by sterile distilled water.

Nodules were then separated from the roots using sterile forceps, and the nodule sap was extracted by gently crushing the nodules. The extracted material was spread onto solid yeast mannitol agar (YMA) medium containing 10 g mannitol, 0.5 g K_2_HPO_4_, 0.2 g MgSO_4_·7H_2_O, 0.1 g NaCl, 0.4 g yeast extract, and 12 g agar per liter, adjusted to around pH 6.8–6.9. The inoculated plates were spread using a sterile microbiological loop and incubated at 29 °C, with growth monitored over time.

Colonies with distinct morphological characteristics were re-isolated on YMA plates by streaking to obtain pure cultures of each microorganism. After purification, the isolates were stored in cryotubes containing 20% glycerol and preserved at −80 °C until further analysis.

### 2.3. Growth Curve and Pathogenicity Test

Growth curves of the isolates were determined in triplicate using an initial pre-inoculum prepared for each bacterial strain. To minimize the influence of dead cells, present in the cultures, bacteria grown on YMA plates were recovered during the exponential growth phase, five days after incubation, by transferring bacterial biomass using a sterile Drigalski spatula.

Bacterial biomass obtained from three culture plates was suspended in 5 mL of sterile Milli-Q water. The turbidity of the pre-inoculum prepared in yeast mannitol liquid medium (YML) was adjusted to an optical density of 0.1 at 600 nm (OD600), which was considered the initial concentration. After preparation, the cultures were incubated under constant agitation in an orbital shaker at 150 rpm and 30 °C.

Cell density was monitored by measuring the optical density at 600 nm immediately after inoculation and every 2 h during incubation. In addition, viable cell counts were determined by plating aliquots collected at the same time points onto YMA medium, followed by colony-forming unit (CFU) counting after incubation.

All isolates were subjected to a pathogenicity screening using blood agar medium (40 g blood agar base per liter of water supplemented with 5% *v/v* bovine blood) to detect potentially pathogenic contaminants. Isolates showing clear hemolytic halos were considered positive and excluded from subsequent analyses.

### 2.4. Molecular Identification and Phylogenetic Analysis

Total genomic DNA was extracted using the TIANamp Genomic DNA Kit (Tiangen Biotech Co., Ltd, Beijing, China) according to the manufacturer’s instructions. DNA integrity was verified by electrophoresis on 1% agarose gel, and DNA the concentration range was from 27–65 ng/uL was determined using a Qubit Fluorometer (Thermo Fisher Scientific, Waltham, MA, USA). 

The 16S rRNA gene was amplified by polymerase chain reaction (PCR) using the primers FD1 (5′-CCGAATTCGTCGACAACAGAGTTTGATCCTGGCTCAG-3′) and RD1 (5′-CCCGGGATCCAAGCTTAAGGAGGTGATCCAGCC-3′) [11]. PCR reactions were carried out using PCRBIO Ultra Mix (PCR Biosystems, Wayne, PA, USA) according to the manufacturer’s recommendations, containing 10 pmol of each primer and 40 ng of template DNA. Amplifications were carried out in a thermal cycler (Applied Biosystems, Waltham, MA, USA) under the following conditions: initial denaturation at 94 °C for 5 min; 35 cycles of denaturation at 94 °C for 30 s, annealing at 56 °C for 40 s, and extension at 72 °C for 90 s; followed by a final extension at 72 °C for 7 min. PCR products were purified using the DNA Clean & Concentrator™-5 kit (Zymo Research, Irvine, CA, USA).

Sequencing of the 16S rRNA gene was performed using an ABI 3130xl Genetic Analyzer (Thermo Fisher Scientific, Waltham, MA, USA). Sequencing reactions were prepared according to the manufacturer’s instructions for the BigDye™ Terminator v3.1 Cycle Sequencing Kit (Thermo Fisher Scientific, Waltham, MA, USA), using the primers FD1, RD1, and the internal primers 8f, 362f, 786f, 907r, and 1203f as described by Inui [12]. Sequences longer than 400 bp with Phred quality scores above 20 were selected using ContGen (a sequence assembly tool) [13]. The resulting sequences in FASTA format were compared with GenBank sequences using BLAST (NCBI, available online: https://blast.ncbi.nlm.nih.gov/, accessed on 10 April 2026) [14] and further analyzed using the Ribosomal Database Project (RDP, release current at the time of analysis; https://rdp.cme.msu.edu/, accessed on 10 April 2026).

### 2.5. Construction of a Phylogenetic Tree

The 16S rRNA gene sequences obtained from the isolates, together with reference sequences from representative species of Burkholderia (n = 22), *Rhizobium* (n = 19), *Bacillus* (n = 23), and *Bradyrhizobium* (n = 23), selected according to the List of Prokaryotic names with Standing in Nomenclature (LPSN) [15] and retrieved from GenBank (https://www.ncbi.nlm.nih.gov/genbank/, accessed on 10 April 2026) [16], were used for phylogenetic analysis. Sequence alignment was performed using MAFFT v7 [17], and alignment quality was verified by manual inspection to confirm positional homology and detect poorly aligned terminal regions. Sequence ends were then trimmed, and external gaps were filled with Ns prior to phylogenetic reconstruction.

Phylogenetic inference was conducted using the Maximum Likelihood method implemented in IQ-TREE v3.1.1 [18], and the consensus tree was visualized and edited in the Interactive Tree of Life (iTOL v7) platform [19].

### 2.6. Solubilization of Zinc, Phosphate, Potassium and IAA Quantification

Zinc solubilization ability was evaluated using minimal salts Tris medium [20] supplemented with 0.1% zinc oxide (ZnO) as an insoluble zinc source. Plates containing the medium were inoculated at three equidistant points on the agar surface with 5 μL aliquots of bacterial suspension adjusted to OD600 = 1.0 for each of the seven isolates, and incubated at 30 °C for seven days. The experiment was conducted with five replicates per isolate and repeated twice, and the results are presented as the mean of the observed values. Colony diameter and solubilization halo were measured using Fiji software v2.9.0 [21], and the solubilization index was calculated using the same procedure described for phosphate solubilization.

Potassium solubilization was evaluated by spotting 5 μL of each bacterial culture onto Petri dishes containing modified NBRIP medium [22] composed of glucose (10 g L^−1^), NaCl (1 g L^−1^), MgSO_4_·7H_2_O (1 g L^−1^), NH_4_Cl (5 g L^−1^), glauconite (0.8 g L^−1^), and agar (15 g L^−1^), with final pH adjusted to 7.2. Plates were incubated at 30 °C for three days. The assay followed the same experimental design, with five replicates per isolate and two independent experimental runs, and results are expressed as mean values.

Phosphate solubilization capacity was determined by inoculating the isolates onto Petri dishes containing phosphate-solubilizing medium composed of glucose (10 g), yeast extract (0.05 g), and agar (15 g). Separately sterilized solutions of CaCl_2_ (10%, 50 mL) and K_2_HPO_4_ (10%, 25 mL) were cooled to 50 °C and mixed with the medium before pouring into 90 × 16 mm Petri dishes. Inoculated plates were incubated at 30 °C for 72 h. Plates were photographed every 24 h, and measurements were taken at 48 h to determine halo area [23]. Images were analyzed using Fiji [24], and the phosphate solubilization index (SI) was calculated according to Pande [24]. This assay was also performed with five replicates per isolate and repeated twice, with results expressed as mean values.

### 2.7. Amplification of the Dinitrogenase Reductase Gene nifH and NodA, NodB, NodC and NodD

Selected bacterial consortia were screened for their potential nitrogen fixation ability by detecting the presence of genes associated with this function using the *nifH* marker gene, which has been widely used to identify the diazotrophic capacity of plant-associated bacteria [25,26,27]. Genomic DNA from seven selected consortia was extracted using the Wizard^®^ Genomic DNA Purification Kit (Promega, Madison, WI, USA). Prior to extraction, inocula were grown in yeast mannitol liquid medium (YML) for 48 h under constant agitation. After incubation, cultures were centrifuged, washed, and resuspended in 50 mM EDTA solution, followed by DNA extraction according to the manufacturer’s instructions with minor modifications. DNA concentration and quality were determined by spectrophotometry using a NanoDrop spectrophotometer (Thermo Fisher Scientific, Waltham, MA, USA).

Amplification of the *nifH* gene was performed using the primers IGK3 and DVV described by Gaby and Buckley [28], which have shown high efficiency for *nifH* amplification compared to other primer sets [27]. PCR reactions were prepared in a final volume of 20 μL containing 4 μL of FirePol^®^ Master Mix 5× (Solis BioDyne, Tartu, Estonia), 0.5 μL of primer DVV (10 μM), 0.5 μL of primer IGK3 (10 μM), 2 μL of template DNA (50 ng μL^−1^), and 13 μL of nuclease-free water. Amplification was performed using the same PCR cycling conditions described previously, and the amplified fragments were visualized on 1.5% agarose gels.

### 2.8. Greenhouse Trials

The experiment was conducted under controlled greenhouse conditions at the Weed Science Laboratory (LAPDA), School of Agricultural and Veterinary Sciences, São Paulo State University (FCAV–UNESP), Brazil. Plastic pots (8 L) were filled with a substrate composed of sand and vermiculite (2:1, *v*/*v*). The substrate was washed and sterilized to remove nutrients and eliminate native microorganisms. Seeds were surface-sterilized with 1% sodium hypochlorite and rinsed three times with sterile water.

The experimental design was completely randomized, with five replicates per treatment, and each pot was considered an experimental unit. The microorganisms used were *Bacillus aerophilus* (BA), *Bacillus inaquosorum* (BI), *Bacillus subtilis* (BS), *Bradyrhizobium yuanmingense* (BdY), *Burkholderia lata* (BkL), *Rhizobium tropici* (RT), and *Bradyrhizobium centrosematis* (BdC, strain SEMIA 6144). Strains were recovered from stock cultures and subjected to growth curve analysis in triplicate to determine the appropriate growth stage for inoculation. A pre-inoculum was prepared and used to establish the main inoculum in yeast mannitol liquid medium (YML). Based on the growth curves, inoculation was performed after 6 h for *Bacillus* spp. and after 24 h for the remaining strains.

Surface-sterilized seeds were immersed in the bacterial cultures for 5 min and then sown at a depth of approximately 5 cm in pots containing sterile substrate. One plant was maintained per pot, and each plant was considered an experimental unit, resulting in five plants evaluated per treatment.

Forty days after seedling emergence, plants were harvested and the following variables were evaluated: chlorophyll content, root length, leaf area, plant height, number of nodules, shoot dry mass, leaf dry mass, root dry mass, and total dry mass.

Data were subjected to analysis of variance (ANOVA) after testing for normality and homogeneity of variances. When assumptions were met, means were grouped using the Scott–Knott test at the 5% significance level.

## 3. Results

### 3.1. Isolation of Microorganisms in Nodules and Pathogenicity Testing

Eight bacterial isolates were obtained, four (A–D) from nodules of plants grown without herbicide application and four (1–4) from nodules of plants grown under herbicide treatment. Pathogenic screening using blood agar indicated that only isolate B produced a clear hemolytic halo, and this isolate was therefore excluded from further analyses.

### 3.2. Molecular Identification of Isolates

**Figure 1:**
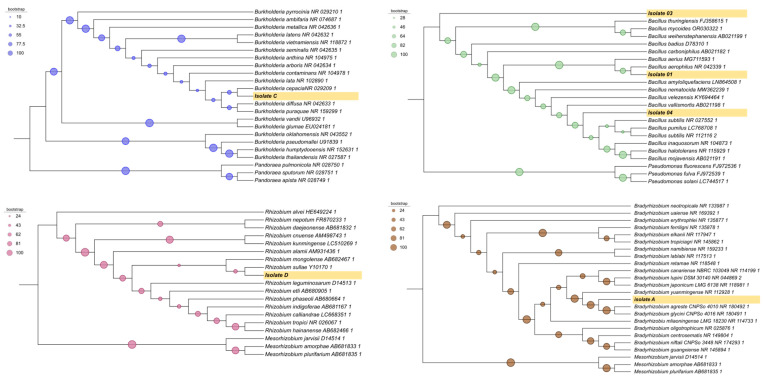
Phylogenetic tree based on 16S rRNA gene sequences of six taxa reconstructed using the Maximum Likelihood method. Bootstrap values are shown at the nodes. The isolates obtained in this study are indicated in bold and highlighted in yellow.

Molecular identification based on 16S rRNA gene sequencing allowed the classification of the isolates as follows: isolate 1 = *Bacillus aerophilus* (GenBank: PP554511); isolate 2 = *Bacillus aerophilus* (GenBank: PP554512); isolate 3 = *Bacillus inaquosorum* (GenBank: PP554513); isolate 4 = *Bacillus subtilis* (GenBank: PP554514); isolate A = *Bradyrhizobium yuanmingense* (GenBank: PP554515); isolate C = *Burkholderia lata* (GenBank: PP554516); and isolate D = *Rhizobium tropici* (GenBank: PP554517) (Figure 1).

Since isolates 1 and 2 were both identified as *Bacillus aerophilus*, subsequent analyses were performed using only isolate 1.

The phylogenetic reconstruction revealed a consistent clustering pattern among the analyzed sequences, with the recovery of monophyletic clades consistent with their taxonomic classification. The taxa of interest clustered within well-defined subclades together with closely related reference sequences, supported by bootstrap values indicating phylogenetic relatedness. The overall topology supported the placement of the target taxa; however, nodes with low bootstrap support should be interpreted cautiously, as they may result from limited phylogenetic signal or alignment ambiguities (Figure 1).

### 3.3. Growth Curve

**Figure 2:**
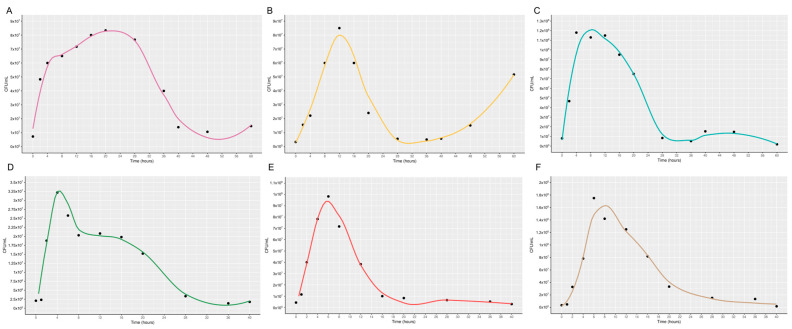
Bacterial growth curve as a function of time and CFU/mL. Black dots indicate specific growth values. Curves smoothed using the LOESS method. (Locally Estimated Scatterplot Smoothing). (**A**) *Bacillus aerophilus*; (**B**) *Bacillus inaquosorum*; (**C**) *Bacillus subtilis*; (**D**) *Bradyrhizobium yanmingense*; (**E**) *Burkholderia lata;* (**F**) *Rhizobium tropici*.

The growth curves of the evaluated strains showed a similar general pattern, characterized by a short lag phase followed by exponential growth, an early population peak, and a subsequent decline in CFU mL^−1^ (Figure 2A–F). However, important differences were observed in growth dynamics and population persistence.

Strains such as *Bacillus aerophilus*, *Bacillus inaquosorum*, and *Burkholderia lata* exhibited rapid initial growth and sharp declines after reaching their population peaks, indicating limited persistence under the tested conditions. In contrast, *Bradyrhizobium yuanmingense* and *Rhizobium tropici* showed a more gradual decline, suggesting greater stability and sustained viability over time.

Additionally, *Bacillus subtilis* displayed intermediate behavior, with rapid growth followed by a progressive but prolonged decrease in population density. Overall, these differences suggest variability in adaptation capacity, resource utilization, and tolerance to stress conditions among the strains.

### 3.4. Solubilization of Zinc, Phosphate, Potassium, Biofilm Production and Quantification of IAA

Evaluation of mineral solubilization activity showed that none of the isolates were able to solubilize potassium or zinc. In contrast, phosphate solubilization was detected in *Bacillus aerophilus*, *Bacillus inaquosorum*, *Bacillus subtilis*, and *Burkholderia lata* (Table 1).

*Burkholderia lata* showed the highest solubilization index among the evaluated isolates, being the only one to exhibit a marked increase in halo size over time (Table 2). The remaining isolates showed lower and more stable solubilization patterns, with no substantial variation between evaluation times.

Biofilm production differed significantly among treatments (F = 12.73, *p* < 0.01), with considerable variability among isolates and their combinations (CV = 34.54%) (Table 3).

Among individual isolates, *Bradyrhizobium centrosematis*, *Bradyrhizobium yuanmingense*, and *Burkholderia lata* showed the highest biofilm production, with no significant differences among them, while *Rhizobium tropici* exhibited markedly lower values. The remaining isolates presented intermediate responses.

Bacterial interactions influenced biofilm formation, with some combinations showing enhanced production compared to individual isolates. Notably, combinations involving *Bacillus* spp. and *Bradyrhizobium centrosematis* tended to increase biofilm formation, whereas other associations resulted in reduced production, suggesting possible incompatibility among specific isolates.

IAA production differed significantly among isolates (F = 101.10, *p* < 0.01), with high experimental precision (CV = 1.75%) (Table 4). *Burkholderia lata* showed the highest IAA production, clearly outperforming the other isolates. Most of the remaining isolates exhibited intermediate and statistically similar values, while *Bradyrhizobium yuanmingense* and especially *Bradyrhizobium centrosematis* showed reduced IAA production, with the latter presenting the lowest values among all isolates.

### 3.5. PCR Amplification of nifH, nodA, nodB, and nodC Genes in the Bacterial Isolates

**Figure 3:**
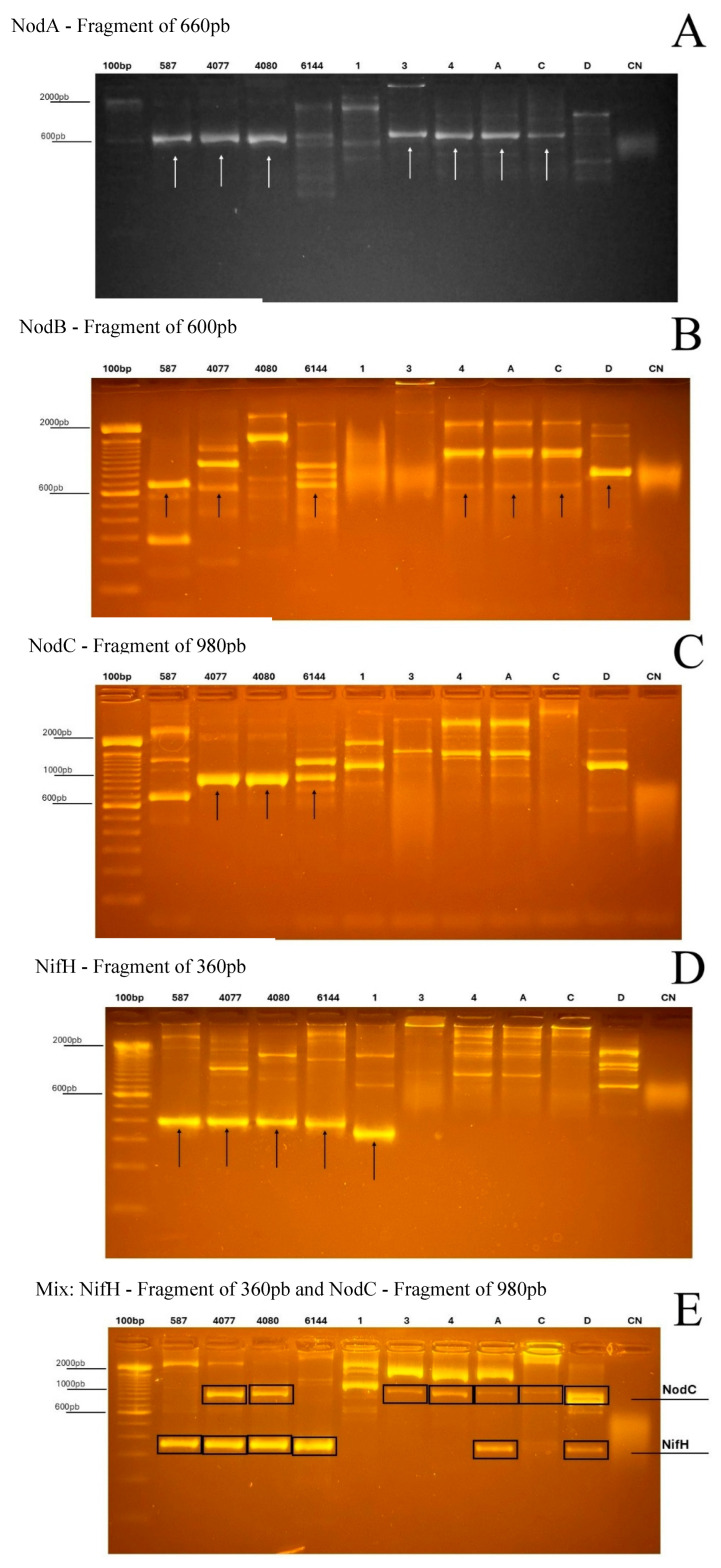
PCR amplification of *nodA*, *nodB*, *nodC*, and *nifH* genes in bacterial isolates obtained from peanut nodules. Agarose gel electrophoresis showing amplicons with sizes consistent with the expected fragments for *nodA* (~660 bp), *nodB* (~600 bp), *nodC* (~980 bp), and *nifH* (~360 bp). Lanes correspond to the molecular marker (M), bacterial isolates, and negative control (CN). Amplicon detection indicates the presence of sequences compatible with the primer sets used, but does not by itself demonstrate gene functionality.

PCR amplification of *nodA*, *nodB*, *nodC*, and *nifH* revealed bands of the expected size in a subset of the isolates (Figure 3), indicating the presence of DNA regions compatible with the corresponding primer sets. However, these results should be interpreted cautiously, as PCR detection alone does not confirm gene integrity, expression, or functionality. Thus, the observed amplicons are considered here as evidence of putative *nod*- and *nifH*-related sequences.

Amplicons consistent with the expected size for *nodA* (~660 bp) were detected in isolates 1 and 2 (*Bacillus aerophilus*), isolate 3 (*Bacillus inaquosorum*), isolate 4 (*Bacillus subtilis*), and in isolates A (*Bradyrhizobium yuanmingense*), C (*Burkholderia lata*), and D (*Rhizobium tropici*). A similar amplification pattern was observed for *nodB* (~600 bp), with bands detected in isolates 1–4 as well as in isolates A, C, and D.

For *nodC* (~980 bp), amplification was observed in isolate 3 (*B. inaquosorum*), isolate 4 (*B. subtilis*), and in isolates A (*B. yuanmingense*), C (*B. lata*), and D (*R. tropici*), whereas isolates 1 and 2 did not show clear amplification with this primer set. This absence may reflect either the lack of the target sequence or sequence divergence in the primer-annealing regions.

The *nifH* gene (~360 bp) showed the broadest distribution among the evaluated isolates, with amplicons detected in isolates 1 and 2 (*B. aerophilus*), isolate 3 (*B. inaquosorum*), isolate 4 (*B. subtilis*), and in isolates A (*B. yuanmingense*), C (*B. lata*), and D (*R. tropici*).

Multiplex PCR targeting *nodC* and *nifH* produced bands consistent with both expected fragments in isolates A, C, and D, whereas the *Bacillus* isolates predominantly yielded the *nifH*-like amplicon and generally lacked a clear *nodC* product. Taken together, these results suggest differences in the distribution of putative symbiosis- and nitrogen fixation-related sequences among the isolates. Nevertheless, confirmation of gene identity and assessment of their biological relevance will require additional analyses, including amplicon sequencing and functional validation.

### 3.6. Greenhouse Trials

**Figure 4:**
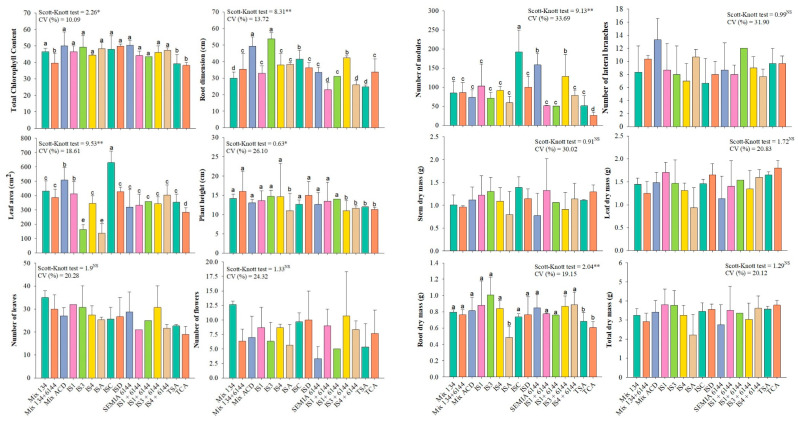
Growth parameters of peanut (*Arachis hypogaea* L., cv. IAC 503) inoculated with different bacterial isolates (IS). Different lowercase letters indicate significant differences (Scott–Knott, *p* ≤ 0.05). * and ** indicate significance at *p* ≤ 0.05 and *p* ≤ 0.01, respectively; NS = not significant.

No significant differences among treatments were observed for shoot dry mass (MSF), stem dry mass (MSC), and total dry mass (MST) (Figure 4). Similarly, chlorophyll content did not differ among treatments, except for plants inoculated with the bacterial mixture (ISA + ISC + ISD) and the fertilized and non-fertilized controls.

Plant height was reduced in plants inoculated with ISA (*Bradyrhizobium yuanmingense*), IS4 (*Bacillus subtilis*) + IS6144 (*Bradyrhizobium centrosematis*), and in the control treatments, while no significant differences were observed among the remaining treatments.

For leaf area, the highest values were observed in plants inoculated with the mixture (ISA + ISC + ISD) and ISC (*Burkholderia lata*), whereas the lowest values occurred in plants inoculated with ISA and IS3 (*Bacillus inaquosorum*).

The most pronounced treatment effects were observed in the root system. Greater root growth was recorded in the presence of the mixture (ISA + ISC + ISD), IS3, and ISC. The highest number of nodules was observed for ISC and IS6144, with ISC showing the strongest effect. Root dry mass (MSR) did not differ among treatments, except for ISA.

## 4. Discussion

Our results indicate that peanut nodules harbor a cultivable bacterial community composed not only of classical symbiotic rhizobia, but also of non-rhizobial endophytes. This finding is consistent with previous reports showing that nodule-associated bacteria may display diverse physiological traits and potential plant growth-promoting functions beyond biological nitrogen fixation [1,2,3,4,5,7,29].

In this context, the isolates obtained in the present study support the concept that nodules represent a structured microbiome in which different taxa may perform complementary functions, contributing to the functional stability of plant–microorganism interactions. 

The taxonomic composition of the cultivable isolates differed between areas with and without herbicide application. Nodules collected from imazapic-treated areas yielded only *Bacillus* isolates, whereas those from untreated areas contained a more diverse assemblage, including *Rhizobium*, *Bradyrhizobium*, and *Burkholderia*. Although the number of isolates examined is limited, this pattern is compatible with the possibility that herbicide management influences the structure of the cultivable nodule-associated bacterial community. Such an interpretation is supported by previous studies showing that herbicides can alter soil microbial composition by disproportionately affecting sensitive taxa and favoring more resilient groups [30,31,32,33]. In this framework, the predominance of *Bacillus* may be related to the well-known ecological resilience of this genus, particularly its capacity for endospore formation and persistence under environmental stress. However, it should be considered that the present study evaluated only the cultivable fraction of the nodule microbiota, and therefore the observed differences reflect changes in the recoverable community rather than in the total microbiome associated with the nodules.

The growth curves revealed clear differences in growth dynamics among the isolates, pointing to distinct physiological strategies that may influence persistence and competitiveness in the rhizosphere and during nodule colonization. *Bacillus subtilis* and *Burkholderia lata* showed rapid biomass accumulation followed by an early decline, a pattern generally associated with copiotrophic-like microorganisms that respond quickly to resource availability but may be less stable under nutrient depletion [34]. By contrast, the slower and more sustained growth of *Rhizobium tropici* is consistent with a more conservative pattern of resource use, often linked to adaptation to nutrient-poor environments [35]. While these ecological interpretations remain inferential, they are consistent with the idea that differences in growth strategy may shape the ability of bacterial taxa to establish and persist in plant-associated niches.

Differences in indole-3-acetic acid (IAA) production among the isolates suggest variation in their plant growth-promoting potential. In particular, the high IAA production observed in *Burkholderia lata* agrees with previous reports identifying members of the Burkholderiaceae as important auxin producers that can modify root architecture and improve nutrient uptake [36,37,38]. By stimulating lateral root and root hair formation, bacterial IAA may contribute to improved nutrient acquisition and stress tolerance in the host plant [39].

Biofilm formation differed among isolates and their combinations, indicating variation in ecological compatibility among the evaluated microorganisms. Greater biofilm production in some consortia may reflect more favorable interactions for persistence, whereas lower production in others may suggest reduced compatibility. Because biofilms enhance protection against environmental fluctuations [40,41], these results are consistent with the view that functional complementarity contributes to the stability of synthetic microbial communities [42].

The detection of *nifH*- and *nod*-related amplicons in both rhizobial and non-rhizobial isolates highlights the genetic diversity associated with the peanut nodule microbiota. While nitrogen fixation and nodulation are typically linked to canonical symbiotic genera such as *Rhizobium* and *Bradyrhizobium*, accumulating evidence indicates that non-rhizobial bacteria may also carry genes associated with nitrogen metabolism or coexist functionally with classical diazotrophs in plant-associated environments [9,43,44]. However, the presence of PCR amplicons alone should not be taken as evidence of active nitrogen fixation or nodulation. Rather, these results indicate the occurrence of sequences compatible with the targeted genes and point to a potentially broader genetic repertoire within the nodule-associated community. Confirmation of their identity, expression, and biological relevance will require further analyses, including amplicon sequencing and functional validation.

Greenhouse assays integrated the in vitro results and demonstrated that the most evident effects of inoculation occurred in the root system, including increases in root length, nodulation, and leaf area in specific treatments. These responses suggest that plant growth promotion is primarily mediated at the soil–root interface through coordinated microbial mechanisms.

In particular, the high production of indole-3-acetic acid (IAA), especially by *Burkholderia lata*, may have contributed to the modulation of root system architecture, stimulating lateral root formation and root hair development, thereby increasing root surface area and enhancing nutrient uptake efficiency [36,37,38,39]. This effect is consistent with the observed increases in root length and leaf area.

In addition, biofilm formation may have played a key role in improving bacterial adhesion, colonization, and persistence in the rhizosphere and within nodules. Biofilms provide protection against environmental fluctuations and facilitate stable plant–microbe interactions, which can enhance root colonization efficiency and nutrient exchange processes [40,41,42].

The observed increase in nodulation may also be associated with improved signaling and infection processes, potentially influenced by interactions between rhizobial and non-rhizobial bacteria carrying nodulation- and nitrogen fixation-related genes [9,43,44]. Such interactions may enhance infection efficiency and nodule establishment, contributing to greater symbiotic performance.

Furthermore, the ability of isolates to solubilize nutrients, particularly phosphate, may have increased nutrient availability in the rhizosphere, supporting root development and overall plant growth. The combination of these traits—hormone production, biofilm formation, and nutrient mobilization—likely explains the enhanced root performance and highlights the importance of multifunctional bacteria in improving nutrient acquisition and tolerance to abiotic stress [45,46,47,48].

Overall, the results indicate that bacteria isolated from peanut nodules form a functionally diverse community with multiple traits associated with plant growth promotion. Herbicide management appears to act as a selective factor, favoring more tolerant taxa such as Bacillus, whereas untreated areas maintain higher microbial diversity. These findings reinforce that the selection of microorganisms for agricultural applications should consider multiple functional attributes rather than single traits, highlighting the role of nodule-associated bacteria in agroecosystem resilience and their potential for biotechnological use.

## 5. Conclusions

This study demonstrates that herbicide management influences the composition of cultivable bacterial communities in peanut nodules, with implications for plant–microbe interactions in the soil environment. The results highlight the functional diversity of nodule-associated bacteria, particularly their roles in modulating root architecture, enhancing nutrient availability through solubilization processes, and improving root colonization via biofilm formation.

Among the evaluated isolates, Burkholderia lata showed the most consistent performance across traits related to plant growth promotion and root development. Its ability to produce indole-3-acetic acid, solubilize nutrients, and form biofilms suggests a key role in regulating rhizosphere processes, including root expansion, nutrient acquisition, and microbial persistence.

These findings indicate that non-rhizobial nodule-associated bacteria can contribute to soil functional processes and plant performance, representing promising candidates for bioinoculant development, particularly in agroecosystems where chemical management may alter microbial community structure.

## Figures and Tables

**Table 1:** Qualitative screening of potassium, zinc, and phosphate solubilization activity in the bacterial isolates.

	Potassium	Zinc	Phosphate
*Bacillus aerophilus*	−	−	+
*Bacillus inaquosorum*	−	−	+
*Bacillus subtilis*	−	−	+
*Bradyrhizobium yuanmingense*	−	−	−
*Bradyrhizobium centrosematis*	−	−	−
*Burkholderia lata*	−	−	+
*Rhizobium tropici*	−	−	+

+ = resultado positivo: presença de halo de hidrólise menor; − = resultado negativo: ausência de halo.

**Table 2:** Solubilization index (SI) for potassium, zinc, and phosphate solubilization by the bacterial isolates.

Isolated	Potassium		Zinc		Phosphate	
	24 h	48 h	24 h	48 h	24 h	48 h
*Bacillus aerophilus*	0	0	0	0	1.42 c	1.42 b
*Bacillus inaquosorum*	0	0	0	0	1.81 b	1.81 b
*Bacillus subtilis*	0	0	0	0	1.29 c	1.29 b
*Bradyrhizobium yuanmingense*	0	0	0	0	0 d	0 c
*Burkholderia lata*	0	0	0	0	2.28 a	3.34 a
*Rhizobium tropici*	0	0	0	0	0 d	0 c
*Bradyrhizobium centrosematis* (6144)	0	0	0	0	0 d	0 c
Causes of variation						
Ftest	-	-	-	-	12.05 **	33.65 **
CV (%)	-	-	-	-	13.14	14.31

Means followed by different lowercase letters within the same column differ significantly according to the Scott–Knott grouping test at the 5% probability level (*p* ≤ 0.05). The symbol ** indicates that the F-test was significant at the 1% probability level (*p* ≤ 0.01).

**Table 3:** Biofilm formation by bacterial isolates evaluated individually and in bacterial consortia.

Isolated	Biofilm Formation (OD_570_)
*Bacillus aerophilus*	1.085 b
*Bacillus inaquosorum*	0.686 c
*Bacillus subtilis*	0.931 b
*Bradyrhizobium yuanmingense*	1.267 a
*Burkholderia lata*	1.221 a
*Rhizobium tropici*	0.045 d
*Bradyrhizobium centrosematis*	1.473 a
BA + BI + BS	1.549 a
BA + BI + BS + BdC	1.420 a
BdY + BkL + RT	0.17 d
BA + BdC	1.236 a
BI + BdC	1.078 b
BS + BdC	0.854 b
BdY + BdC	1.120 b
BkL + BdC	0.415 c
RT + BdC	0.022 d
Causes of variation	
Ftest	12.73 **
CV (%)	34.54

Means followed by different lowercase letters within the same column differ significantly according to the Scott–Knott grouping test at the 5% probability level (*p* ≤ 0.05). The symbol ** indicates that the F-test was significant at the 1% probability level (*p* ≤ 0.01).

**Table 4:** Quantification of indole-3-acetic acid (IAA) production by the bacterial isolates (µg mL^−1^).

Isolated	µg mL^−1^
*Bacillus aerophilus*	6.80 b
*Bacillus inaquosorum*	6.57 c
*Bacillus subtilis*	6.65 b
*Bradyrhizobium yuanmingense*	6.46 c
*Burkholderia lata*	7.93 a
*Rhizobium tropici*	6.80 b
*Bradyrhizobium centrosematis* (6144)	5.30 d
Causes of variation	
Ftest	101.10 **
CV (%)	1.75

Means followed by different lowercase letters within the same column differ significantly according to the Scott–Knott grouping test at the 5% probability level (*p* ≤ 0.05). The symbol ** indicates that the F-test was significant at the 1% probability level (*p* ≤ 0.01).

## Data Availability

The data that support the findings of this study are available from the corresponding author upon request.
